# Validation of precision-cut liver slices to study drug-induced cholestasis: a transcriptomics approach

**DOI:** 10.1007/s00204-016-1778-8

**Published:** 2016-06-25

**Authors:** Suresh Vatakuti, Peter Olinga, Jeroen L. A. Pennings, Geny M. M. Groothuis

**Affiliations:** 1 Division of Pharmacokinetics, Toxicology and Targeting, Groningen Research Institute for Pharmacy, University of Groningen, Antonius Deusinglaan 1, 9713 AV Groningen, The Netherlands; 2 Division of Pharmaceutical Technology and Biopharmacy, Groningen Research Institute for Pharmacy, University of Groningen, Groningen, The Netherlands; 3 National Institute for Public Health and the Environment, Bilthoven, The Netherlands

**Keywords:** Cholestasis, Hepatotoxicity, Precision-cut liver slices, Transcriptomics

## Abstract

Hepatotoxicity is one of the major reasons for withdrawal of drugs from the market. Therefore, there is a need to screen new drugs for hepatotoxicity in humans at an earlier stage. The aim of this study was to validate human precision-cut liver slices (PCLS) as an ex vivo model to predict drug-induced cholestasis and identify the possible mechanisms of cholestasis-induced toxicity using gene expression profiles. Five hepatotoxicants, which are known to induce cholestasis (alpha-naphthyl isothiocyanate, chlorpromazine, cyclosporine, ethinyl estradiol and methyl testosterone) were used at concentrations inducing low (<30 %) and medium (30–50 %) toxicity, based on ATP content. Human PCLS were incubated with the drugs in the presence of a non-toxic concentration (60 µM) of a bile acid mixture (portal vein concentration and composition) as model for bile acid-induced cholestasis. Regulated genes include bile acid transporters and cholesterol transporters. Pathway analysis revealed that hepatic cholestasis was among the top ten regulated pathways, and signaling pathways such as farnesoid X receptor- and liver X receptor-mediated responses, which are known to play a role in cholestasis, were significantly affected by all cholestatic compounds. Other significantly affected pathways include unfolded protein response and protein ubiquitination implicating the role of endoplasmic reticulum stress. This study shows that human PCLS incubated in the presence of a physiological bile acid mixture correctly reflect the pathways affected in drug-induced cholestasis in the human liver. In the future, this human PCLS model can be used to identify cholestatic adverse drug reactions of new chemical entities.

**Electronic supplementary material:**

The online version of this article (doi:10.1007/s00204-016-1778-8) contains supplementary material, which is available to authorized users.

## Introduction

Drug-induced hepatotoxicity (DILI) is one of the major reasons for failure of drugs in the drug development or post-marketing phase, leading to withdrawal of drugs from development or from the market, respectively. It is of major concern for the consumers, regulatory authorities such as the FDA and the EMA and pharmaceutical companies. In the drug discovery process, valuable information about possible mechanisms of toxicity is gained by exposing a compound to suitable ex vivo, in vitro or in vivo models. The possibility to determine early in the drug development process whether a compound causes a particular pathology results in valuable information about the mechanism of action of an as yet uncharacterized compound.

One of the major causes for DILI is cholestasis. Cholestasis is characterized as inhibition of bile flow caused by a variety of mechanisms that can involve not only elements of the biliary tree, including the bile ducts and ductules, but also the transporters in the basolateral or canalicular membrane, as well as disruption of the tight junctions of the hepatocytes. BSEP inhibition is a common cause of cholestasis, and the resulting accumulation of bile acids in the hepatocytes triggers a direct cellular response, which is associated with apoptosis, inflammation, oxidative stress and endoplasmic reticulum stress and cell death. It also causes adaptive cellular responses mediated via nuclear receptors (Vinken et al. 2013).

Most of the information has been obtained from in vivo models such as bile duct ligation in different animal species, but mechanistic information on human liver in vivo is very scarce. In addition, in vitro models such as human and rat hepatocytes, HepG2 and HepaRG cells have been investigated as predictive model. Ansede et al. (2010) published an in vitro assay to assess the transporter-based inhibition of excretion of a tracer concentration of bile acid using sandwich-cultured rat hepatocytes. But as bile acids are only present at very low concentration, this model may not detect bile acid-dependent toxicity. In contrast, Ogimura et al. (2011) incubated sandwich-cultured rat hepatocytes with cholestatic compounds in the presence of bile acids to develop an experimental model reflecting bile acid-dependent cholestatic injury. Both models were developed with rat hepatocytes, and extrapolation of the results to predict human cholestatic injury remains hazardous. Recently, Chatterjee et al. (2014) showed that indeed a model with sandwich-cultured human hepatocytes in the presence of bile acids predicts the cholestatic potential of drugs more accurately in human than rat hepatocytes. However, it is well known that the hepatocytes show dedifferentiation during culture, which is accompanied by a decrease in drug metabolism capacity and unphysiological expression of transporters (Fraczek et al. 2013). Therefore, predictions based on sandwich cultures may not always be very precise.

In the present study, we aimed to investigate whether human precision-cut liver slices (PCLS) in the presence of a physiological concentration of bile acids could be used as a predictive model for drug-induced bile acid-dependent cholestatic injury. PCLS have been shown to be a viable ex vivo tool to study the metabolism and toxicity of xenobiotics for over a decade (de Graaf et al. 2010). Recently, mouse PCLS were used as a model to identify the mechanisms of drug-induced cholestasis (Szalowska et al. 2013), but also, this mouse model is prone to problems of species extrapolation and no bile acids were present during the incubation. The possibility to use human tissue for toxicity studies helps to reduce unnecessary animal studies and to identify human specific toxicity. Advantages of this PCLS model include the presence of all cells of the tissue in their natural environment with intact inter-cellular and cell–matrix interactions, stable expression of drug metabolizing and detoxification enzymes, the ability to produce bile acids and most importantly polarized physiological expression of transporters. This model is therefore highly appropriate for studying multicellular drug toxicity processes (van de Bovenkamp et al. 2005; Elferink et al. 2008, 2011; Hadi et al. 2013; Westra et al. 2014; Vatakuti et al. 2015). Elferink et al. (2008) used transcriptomics analysis to show that rat PCLS reflect the proper mechanisms of hepatotoxicity. Moreover, they showed that in human PCLS the gene expression profiles of genes related to drug metabolism, transport and toxicity remain fairly constant during 24 h of incubation but that these profiles are affected by paracetamol-induced toxicity. However, up to now, no transcriptomics data on human PCLS exposed to cholestatic compounds have been reported. Since the primary event involved in the majority of drug-induced cholestasis in vivo is accumulation of bile acids due to inhibition of export transporters such as BSEP, we hypothesized that to further optimize the PCLS to mimic cholestasis induced by accumulation of the bile acids in vivo, the PCLS should be incubated with a non-toxic concentration of a bile acid mix mimicking the portal vein bile acid concentration and composition. Therefore, in this study, we exposed human PCLS to drugs or chemicals known to induce cholestasis such as alpha-naphthyl isothiocyanate (ANIT), cyclosporine, chlorpromazine, ethinyl estradiol and methyl testosterone at different concentrations inducing low-to-moderate toxicity based on decrease in ATP content in the presence of a physiological bile acid mix. To detect cholestatic injury and the mechanisms involved, the profiles of differentially expressed genes were analyzed using IPA pathway analysis. Moreover, they were compared to expression profiles of human in vivo late-stage cholestasis caused by biliary atresia and intrahepatic cholestasis.

## Materials and methods

### Chemicals

Alpha-naphthylisothiocyanate (ANIT), chlorpromazine (CP), cyclosporine (CS), ethinyl estradiol (EE) and methyl testosterone (MT) were purchased from Sigma-Aldrich (St. Louis, MO, USA). Stock solutions for all compounds were prepared in DMSO (VWR, Briare, France).

### Human liver tissue

Human liver tissue was obtained from the remaining liver tissue after split liver transplantation (TX). The characteristics of the human livers used in the experiments are described in Table 1. The experimental protocols were approved by the Medical Ethical Committee of the University Medical Center, Groningen.

**Table 1:** Demographics of donors of human liver tissue used for the experiments

Human liver	Sex	Age
1	Female	58
2	Male	50
3	Female	71
4	Male	24
5	Female	24
6	Male	64

### Preparation and incubation of human PCLS

Precision-cut liver slices of 5 mm diameter and 250 μm thickness were prepared (de Graaf et al. 2010). At this thickness, full penetration of substrates has been shown. PCLS were made using the Krumdieck tissue slicer (Alabama R&D, Munford, AL, USA) in ice-cold Krebs buffer at pH 7.42, enriched with glucose to a final concentration of 25 mM, saturated with carbogen (95 % O_2_/5 % CO_2_). Immediately after the slices were made, they were placed in ice-cold University of Wisconsin organ preservation solution (UW, Dupont Critical Care, Waukegan, IL, USA) and stored on ice until the beginning of the experiment. Slices were pre-incubated individually in 12-well plates in 1.3 ml of Williams Medium E with glutamax-1 (Gibco, Invitrogen, Paisley, Scotland) supplemented with 25 mM d-glucose and 50 μg/ml gentamycin (Gibco, Invitrogen, Paisley, Scotland). In the incubator (Sanyo CO_2_/O_2_ Incubator, PANASONIC, Secaucus, NJ, USA), the plates were gently shaken (90 times/min) for 1 h under 80 % O_2_ and 5 % CO_2_ atmosphere at 37 °C. This pre-incubation allows the slices to restore their ATP levels. After pre-incubation, the slices were moved to different well plates filled with 1.3 ml Williams Medium E with glutamax-1 supplemented with 25 mM d-glucose, 50 μg/ml gentamycin, 60 μM human bile acid mix (Table 2) and different concentrations of the compounds such as ANIT (25, 50 and 75 μM), chlorpromazine (10, 20 and 30 μM), cyclosporine (9, 12 and 15 μM), ethinyl estradiol (25, 50 μM and 75 μM) and methyl testosterone (50, 75 and 100 μM) or solvent (DMSO). These concentrations were selected from pilot experiments where a larger concentration range was used to detect concentrations that resulted in low-to-medium toxicity. The plates were incubated under the same conditions for 24 h. All incubation conditions were carried out in triplicate. Each experiment was performed in slices of 6 different human livers (Table 1). Slices were harvested for ATP assay in 100 mM Tris buffer with 2 mM EDTA and for RNA isolation in RNAlater. The bile acid mix was added to the medium in order to create an environment similar to the physiological concentration in the portal vein. The composition of the serum bile acids was according to Scherer et al. (2009). Pilot experiments were performed to find out the non-toxic concentration of the bile acid mix. A series of concentrations (10–200 μM) of the bile acid mix containing the 14 different bile acids (BA) shown in Table 2 were tested. Concentrations up to 60 μM were found to be non-toxic, and hence, further experiments were performed using a concentration of 60 μM, which is close to the reported portal vein bile acid concentration (Scherer et al. 2009). The final concentrations of the bile acids in the incubation medium are presented in Table 2.

**Table 2:** Composition of human bile acids mix

Composition of bile acids	Final concentration in the incubation medium (µM)
Cholic acid (CA)	2.65
Chenodeoxycholic acid (CDCA)	4.51
Deoxycholic acid (DCA)	6.37
Glycochenodeoxycholic acid (GCDCA)	22.69
Glycocholic acid (GCA)	5.44
Glycodeoxycholic acid (GDCA)	5.04
Glycoursodeoxycholic acid (GUDCA)	3.72
Hyodeoxycholic acid (HDCA)	2.79
Lithocholic acid (LCA)	0.40
Taurocholic acid (TCA)	0.64
Taurochenodeoxycholic acid (TCDCA)	2.79
Taurolithocholic acid (TLCA)	1.15
Taurodeoxycholic acid (TDCA)	0.58
Ursodeoxycholic acid (UDCA)	1.46

### Viability assay: ATP and protein content of PCLS

The viability of PCLS was assessed by the content of ATP. The determination of ATP was performed using the ATP Bioluminescence Assay Kit CLS II (Roche, Mannheim, Germany). Three slices were harvested in a 2 mM EDTA solution containing 70 % ethanol, pH 10.9, and immediately frozen at −80 °C. Slices were homogenized using a mini beat beater, and the homogenate was centrifuged for 5 min at 13,000*g*. The supernatant was used for the ATP assay and the pellet for the protein analysis. The ATP assay is performed in 96-well plate; 5 μL of sample was diluted 10 times with 100 mM Tris–HCl, 2 mM EDTA buffer pH 7.8; and 50 μL of luciferase was added to each sample, and the ATP was measured with the Lucy1 luminometer (Anthos, Durham, NC, USA). The protein content of each slice was assessed using the Bio-Rad DC protein assay kit (Bio-Rad, Munich, Germany) as described before (de Graaf et al. 2010), and the ATP values are corrected with their corresponding protein content, which shows some variation, due to the presence of large blood vessels and small variations in the diameter between individual slices, but does not change during 24-h incubation (unpublished observations). As shown before (de Graaf et al. 2010), different human livers have different basal ATP levels. Data normalization was performed to account for the inter-individual differences. The response (decrease in ATP) to each of the tested concentrations of the cholestatic compounds was expressed relative to the corresponding control ATP values of PCLS of the same donor incubated under identical conditions but without the drug.

### RNA isolation

RNA was isolated from slices with <10, <30 and 30–50 % decreased viability. Maxwell^®^ 16 LEV Total RNA purification kit (Promega, The Netherlands) with Maxwell^®^ 16 LEV Instrument was used to isolate RNA from the samples. Immediately after isolation, the RNA quality was assessed by measuring the 260/280 and 260/230 ratios, and the concentration was measured with the ND-1000 spectrophotometer (Fisher Scientific, Landsmeer, The Netherlands). The quality (RIN value) and quantity of the RNA were determined by high-throughput Caliper GX LabChip RNA kit (Caliper).

### Amplification, labeling and hybridization of RNA samples

Ambion Illumina Total Prep RNA kit was used to transcribe 300 ng RNA to cRNA according to the manufacturer’s instructions. A total of 750 ng of cRNA was hybridized at 58 °C for 16 h to the Illumina HumanHT-12 v4 Expression BeadChips. BeadChips were scanned using Iscan software, and IDAT files were generated (Illumina, San Diego, CA, USA).

### Preprocessing of gene expression data

GenomeStudio software (Illumina) was used to read the IDAT files and generate raw expression values. The ArrayAnalysis web service (www.arrayanalysis.org/) was used for further preprocessing the data, which uses lumi R package (Eijssen et al. 2013). Raw gene expression data were background-corrected (bgAdjust), variance-stabilized (VST) and normalized by quantile normalization. After normalization, the data were corrected for batch differences owing to RNA isolation and hybridization using the ComBat method implemented in the swamp package in R (Lauss et al. 2013). Differentially expressed genes in slices exposed to the cholestatic drugs in the presence of bile acids versus the slices exposed to vehicle (DMSO) in the presence of bile acids were identified using the unpaired moderated *t* test in the limma package (Ritchie et al. 2015). Genes that are regulated with a criterion of fold change of 1.5 (≤ or ≥1.5), and FDR-corrected *p* value ≤0.05 (Benjamini and Hochberg method) were chosen for pathway analysis. To compare our data with the scarcely available human in vivo data, human in vivo late-stage cholestasis data were downloaded from the Gene Expression Omnibus database (GSE46960). These gene expression data were generated in GeneChip Human Gene 1.0 ST array (Affymetrix, CA), hybridization experiments using human liver biopsies obtained from 64 infants with biliary atresia, 14 age-matched infants with cholestasis of other origin than biliary atresia and from 7 deceased healthy children (Bessho et al. 2014). Affymetrix data normalization and statistical analysis were performed using the ArrayAnalysis Web site (Eijssen et al. 2013) using the same criteria as for the PCLS.

A gene is considered as regulated in association with cholestasis in human PCLS if its expression is differentially regulated in the same direction by two or more of the five tested compounds. A gene is considered as regulated in vivo in human if its expression is differentially regulated in either biliary atresia or intrahepatic cholestasis.

### Pathway analysis

Canonical metabolic and signaling pathway analysis was performed using QIAGEN’s Ingenuity^®^ Pathway Analysis (IPA^®^, QIAGEN Redwood City, California, USA). The compound exposures where no or very few genes were regulated (25 µM AN, 10 and 20 µM CP 25 and 50 µM EE and 25 µM MT) were excluded from the pathway analysis. Comparison pathway analysis feature in IPA was used to compare the canonical pathways affected by the different compounds in human PCLS.

## Results

In this study, human PCLS were pre-incubated for 1 h to restore their ATP levels after storage and processing and subsequently incubated for 24 h in the presence of a physiological concentration of bile acids and in the presence and absence of various concentrations of well-known cholestatic compounds. Transcriptomics analysis was performed on samples showing low (<30 %) or medium (30–50 %) decrease in viability, to understand the mechanistic events involved in drug-induced cholestasis. Differentially regulated genes were identified, and pathway analysis was performed to understand the mechanistic role of the regulated genes as described below.

### Concentration selection for transcriptomic studies

**Fig. 1:**
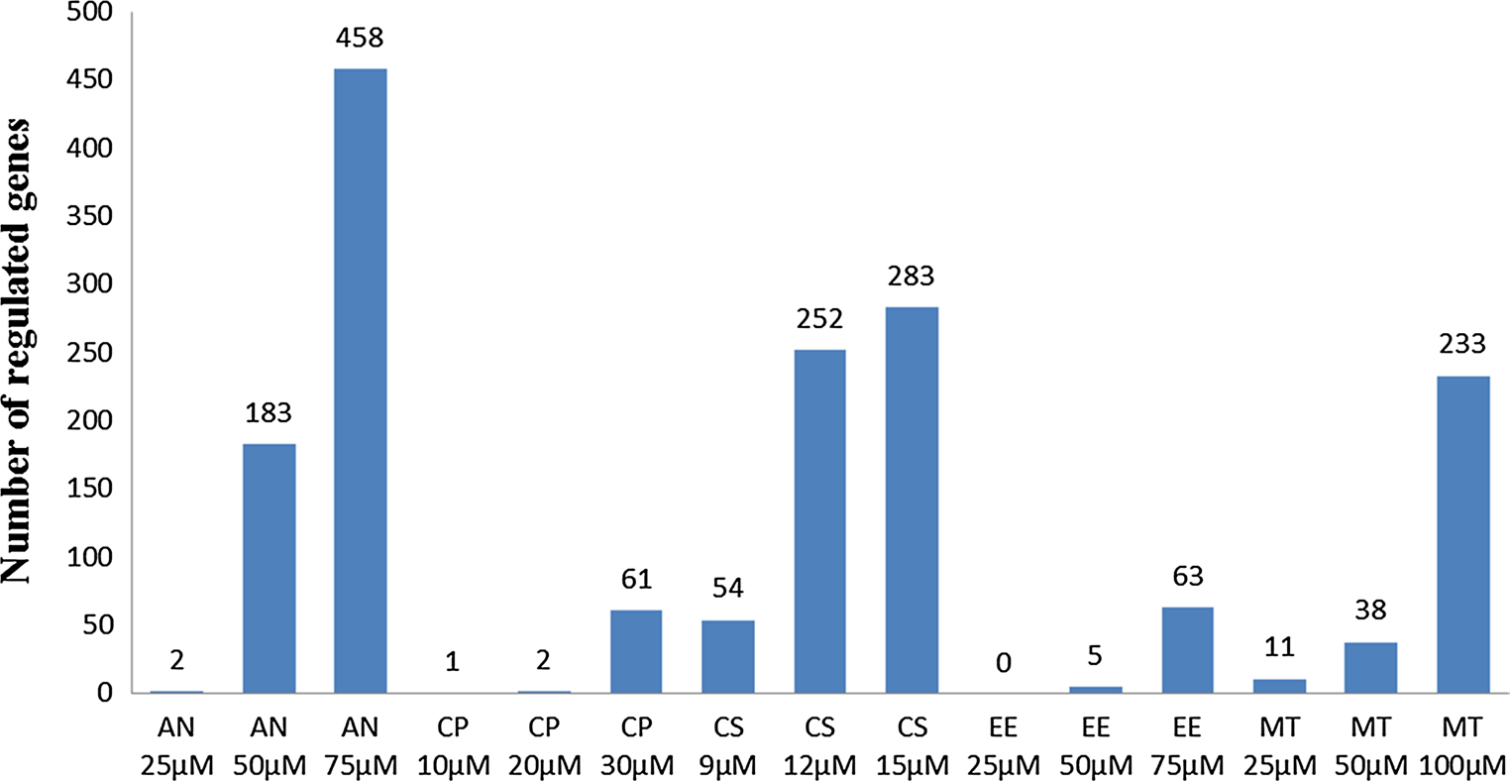
Number of genes differentially regulated with a fold change of 1.5 and multiple hypothesis-adjusted *p* value 0.05

Concentration response studies were performed to identify toxic concentrations for the cholestatic compounds. Pilot studies were performed using a range of concentrations (data not shown), and the concentrations which showed a 10–30 and 30–50 % decrease in ATP for each of the tested cholestatic compounds were chosen for the microarray gene expression studies. All five cholestatic compounds showed a concentration-dependent decrease in cell viability (supplementary data Figure 1). Despite some inter-individual differences in sensitivity for the drugs, for each human liver, the concentrations that caused low (<30 % decrease in ATP) and medium toxicity (30–50 % decrease in ATP) were similar. A concentration-dependent increase in the number of regulated genes was observed. From the data on the number of regulated genes (Fig. 1), it is clear that concentrations that do not result in a substantial reduction in viability do not cause regulation of a significant number of genes. At concentrations causing up to 30 % decrease in viability, a relatively limited number of genes were regulated, and at higher concentrations, where toxicity amounted to 30–50 %, a significant number of genes was regulated.

However, despite of a similar decrease in toxicity, the compounds have different effects on gene expression judged on the basis of a different number of regulated genes. In general, the large majority of genes regulated at the low concentrations were also regulated at the higher concentrations for each of the compounds (results not shown). Between the different compounds, similarities and differences were observed (see supplementary figure 2), which is not surprising as the tested compounds are known to cause cholestasis by different mechanisms. Further pathway analysis of the regulated genes was performed to show overlap in regulated pathways and identify their potential relation with cholestasis (see below).

### Canonical signaling pathway analysis

**Fig. 2:**
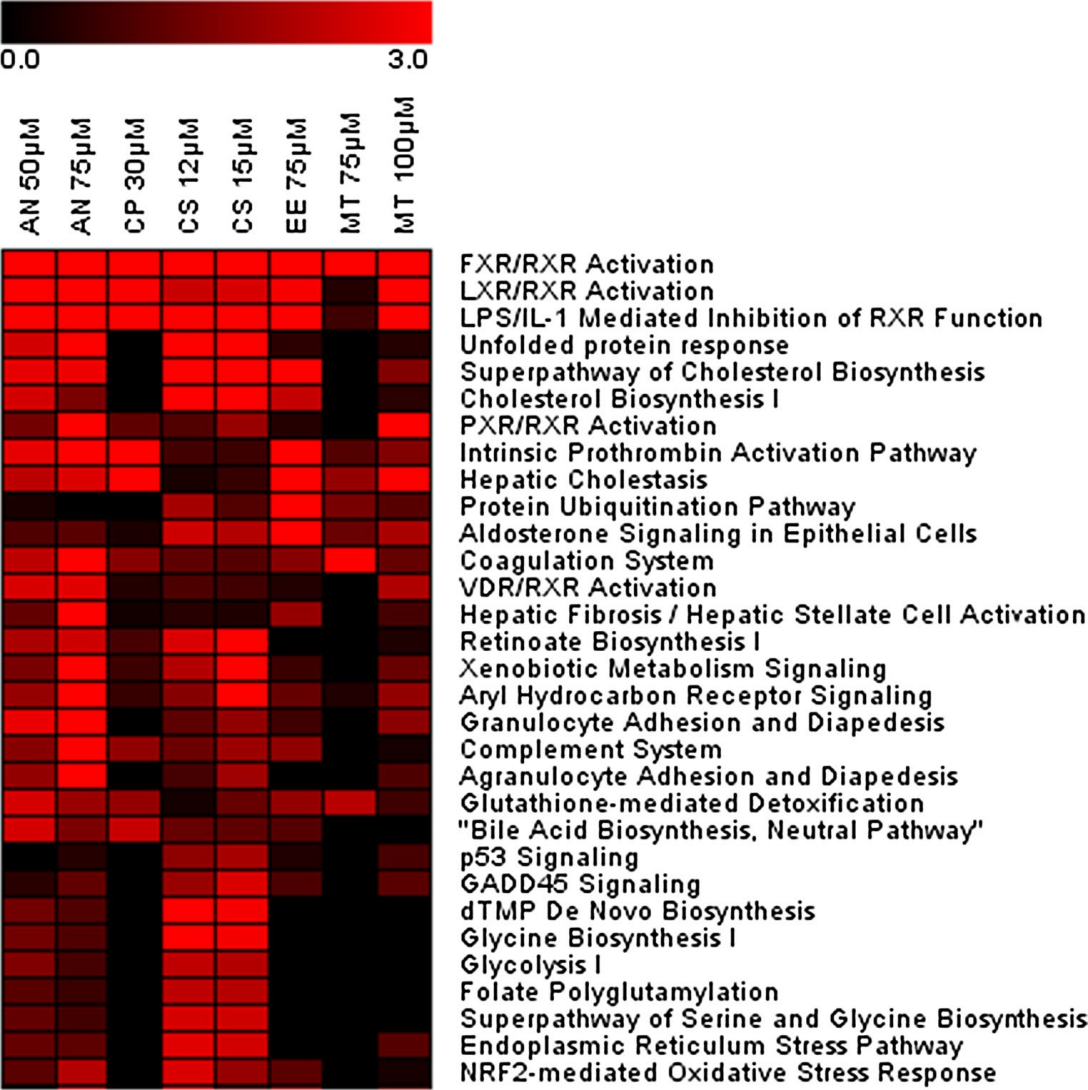
Heatmap of canonical pathway enrichment analysis results. Enrichment values [−log (*p* value)] are scaled from 0 to 3 (*black to red*). Compound exposures where no or very few genes were regulated were excluded (color figure online)

The Ingenuity Knowledge Base contains canonical pathways that are well-characterized metabolic and cell signaling pathways. The differentially regulated genes as shown in Fig. 1 were scored against the canonical pathways, and the resulting scaled values for pathway enrichment are shown as a heatmap in Fig. 2.

Figure 2 shows that many canonical pathways that are related to bile acid homeostasis and cholestasis are regulated in the PCLS treated with the 5 cholestatic compounds in the presence of a portal vein concentration of a physiological bile acid mix. Hepatic cholestasis appeared as one of the top 10 most significantly affected pathways. Moreover, signaling pathways such as FXR, LXR, PXR and VDR, which play a prominent role in cholestasis, are also significantly affected. In addition, endoplasmic reticulum stress (ER stress), unfolded protein response and protein ubiquitination pathways, known to play a role in bile acid-induced cholestasis process (Adachi et al. 2014), are significantly affected. Furthermore, the pathways involved in bile acid-induced damage such as Nrf2-mediated stress response, coagulation system and complement activation appear affected. The observed patterns of activated genes appeared concentration dependent; nevertheless, there was a good overlap of the regulated genes at the different concentrations. Therefore, for the comparison of the genes regulated in these different canonical pathways, the highest concentration for each of the different cholestatic compounds was considered for further analysis. The genes that were regulated significantly by at least one of the compounds in these different pathways are given in Tables 3, 4 and 5 and in the supplementary Tables 1–8, where downregulated genes are highlighted in blue and upregulated genes in orange.

**Table 3:**
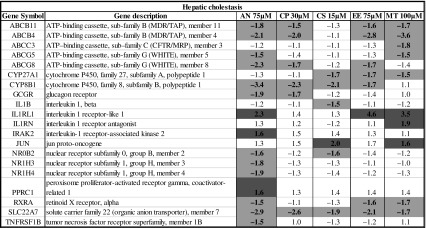
Genes involved in hepatic cholestasis pathway and their regulation after exposure to cholestatic drugs in human PCLS (color figure online)

Significantly regulated genes with fold change ≤−1.5 or ≥1.5 are highlighted in orange and blue, respectively

**Table 4:**
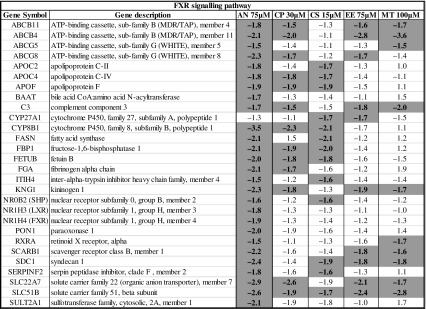
Genes involved in the FXR pathway and their regulation after exposure to cholestatic drugs in human PCLS (color figure online)

Significantly regulated genes with fold change ≤−1.5 or ≥1.5 are highlighted in orange and blue, respectively

**Table 5:**
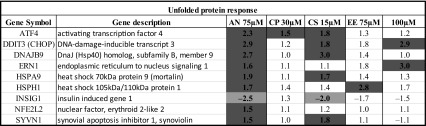
Genes involved in unfolded protein response (UPR) and their regulation after exposure to cholestatic drugs in human PCLS (color figure online)

Significantly regulated genes with fold change ≤−1.5 or ≥1.5 are highlighted in orange and blue, respectively

It is interesting to observe that the genes coding for transporters and metabolic enzymes in the hepatic cholestasis pathway are downregulated (Table 3). It should be mentioned here that most of the genes involved in the hepatic cholestasis pathway are also part of the signaling pathways mentioned below.

The FXR pathway was the most significantly regulated pathway. The genes regulated in the human PCLS that are involved in FXR-mediated response are summarized in Table 4. It is apparent that many target genes involved in the FXR pathway such as BSEP, MRP2, MDR3, BACS, BAAT, FGF15 and PXR are downregulated. The LXR pathway was also significantly downregulated in human PCLS exposed to cholestatic compounds and bile acids. The genes regulated in human PCLS that are involved in LXR-mediated response are summarized in supplementary Table 1. Also, the genes involved in the cholesterol biosynthesis pathway were downregulated in human PCLS by the cholestatic compounds except for CP (supplementary Table 2). The downregulation of the cholesterol biosynthesis genes is well in line with the downregulation of the LXR target genes. As expected, also target genes in the PXR (supplementary Table 3) and VDR (supplementary Table 4) pathways were regulated. Among the PXR target genes, some of the cytochrome P450 isoforms were upregulated ex vivo in the slices. In line with the published involvement of ER stress in cholestasis, the unfolded protein response pathway was significantly regulated and most of the genes involved in unfolded protein response (ER stress) were significantly upregulated (Table 5).

Complement and coagulation pathways were affected, and genes involved in those pathways were mostly downregulated as shown in supplementary Tables 5 and 6, respectively. NRF2-mediated oxidative stress response was affected, and upregulation of genes coding for heat shock proteins and the activation of transcription factors such as ATF4 and NRF2 were apparent (supplementary Table 7). In addition, the hepatic fibrosis pathway was affected, and some genes involved in the hepatic stellate cell activation were upregulated such as KLF6 (supplementary Table 8).

## Discussion

In this study, human PCLS were validated as an ex vivo model that reflects the drug-induced cholestasis processes using transcriptomic analysis. Hepatotoxicants that are known to induce cholestasis in human, such as cyclosporine, chlorpromazine, ethinyl estradiol and methyl testosterone, clearly induced regulation of genes and pathways associated with cholestasis in human PCLS when incubated in the presence of bile acids. In addition, ANIT, a well-known cholestatic compound in rats, was included in the study, and showed a similar cholestatic gene expression pattern in human liver slices. From the data, it is clear that concentrations that do not cause a decrease in viability also do not lead to differential expression of a substantial number of genes. Also, the addition of 60 µM of bile acid mix to the incubation medium does not induce major changes in gene expression (data not shown). Pathway analysis clearly showed a gene expression pattern of cholestatic injury, which was concentration dependent for all drugs (Fig. 1 and supplementary Figure 1). The direction of regulation of most genes was similar among the five tested cholestatic drugs, but there were differences with respect to the number of genes that were significantly regulated in each pathway. Importantly, hepatic cholestasis was among the top 5 regulated pathways. The majority of the pathways regulated in the human PCLS are represented in the adverse outcome pathway (AOP) for cholestasis as proposed by Vinken et al. (2013), including the primary direct cellular responses and secondary adaptive responses involved in bile acid-induced cholestatic injury, such as primary toxicity by NRF2-mediated oxidative stress response, inflammation-mediated hepatic fibrosis, endoplasmic reticulum stress and activation of the coagulation and complement system. Moreover, signaling pathways such as FXR, LXR, PXR and VDR as well as the related cholesterol biosynthesis pathways were affected. The significantly regulated pathways are clearly distinct from the pathways regulated by paracetamol as published by us (Elferink et al. 2011). Moreover, the gene expression pattern obtained by these cholestatic compounds was clearly distinct from those obtained after treatment of PCLS of the same human livers with necrotic compounds, which allowed us to develop a classification model and identify some classifier genes (Vatakuti et al. 2016). Together this indicates that the observed regulated pathways are at least to a large extent related to the cholestatic properties of the tested compounds. Further research with a higher number of human liver samples and more cholestatic drugs will be needed to identify key signature genes that best represent the key events during cholestasis and can identify compounds with cholestatic potential.

Since only the highest toxic concentration tested in our study induced significant gene expression changes related to cholestasis, we compared those concentrations with *C*_max_ values in adult patients after a therapeutic dose in the systemic blood, being 0.42 µM for chlorpromazine, 3.2 µM for cyclosporine, 8.2 nM for ethinylestradiol and 0.13 µM for methyl testosterone. The toxic concentration used in our PCLS study was quite well in the same range as the *C*_max_ for chlorpromazine (~70× *C*_max_), and cyclosporine (~3× *C*_max_), but for ethinylestradiol (~9000× *C*_max_) and methyl testosterone (~770× *C*_max_), much higher concentrations were used ex vivo. *C*_max_ values could not be found for ANIT as it is studied so far only in animals. Although for some compounds we could only see evident changes at high concentrations, this is not unusual and the concentrations tested were similar to the concentrations tested in human hepatocytes as published in the TG-GATEs database (http://toxico.nibiohn.go.jp/open-tggates/english/search.html).

It is generally assumed that the accumulation of bile acids, cholesterol and bilirubin during onset and progression of cholestatic condition induces response processes characterized by the activation of nuclear receptors such as FXR, LXR, PXR and VDR, which triggers cellular adaption to counteract bile acid accumulation and thus cholestatic liver injury (Zollner and Trauner 2008; Wagner et al. 2009). The farsenoid X receptor (FXR) acts as a sensor for intracellular bile acid levels (Makishima et al. 1999), and activation of FXR (NR1H4) induces adaptive gene expression changes in response to accumulation of bile acids in cholestasis such as inhibition of bile acid synthesis, upregulation of phase I bile acid hydroxylation, phase II conjugation enzymes, and induction of the expression of canalicular and basolateral bile acid transporters. In contrast with this expected activation of FXR, in the human PCLS, the target genes in the FXR pathway were downregulated. Interestingly, a similar trend of downregulation of FXR target genes was observed in mouse PCLS after exposure to cholestatic drugs in the absence of bile acids (Szalowska et al. 2013). Important genes known to play a role in cholestasis such as ABCB4 (MDR3), ABCB11 (BSEP) and NR0B2 (SHP) were downregulated in human PCLS exposed to bile acids and cholestatic drugs. Previously, we showed upregulation of SHP and downregulation of BSEP as a response to accumulation of bile acids after incubation of human PCLS with 100 μM CDCA (Jung et al. 2007). Either a limited increase in the concentration of the total bile acids particularly of CDCA in the hepatocytes or the presence of other bile acids such as LCA that can counteract the effect of CDCA (Yu et al. 2002) may be the cause for this difference. Lew et al. (2004) showed that FXR controls the gene expression of its target genes in a ligand-dependent fashion based on the individual bile acids. It is possible that FXR downregulation is due to combined effect of mixture of bile acids used in our experiments instead of using only an FXR agonist such as CDCA reported in other PCLS studies where FXR target genes were upregulated (BSEP, SHP) (Jung et al. 2007). Consistent with our data is the findings that antagonizing FXR activation is a mechanism for lithocholic acid-induced liver toxicity (Yu et al. 2002), whereas the synthetic FXR agonist GW4064 prevents intra- and extrahepatic cholestatic injury (Liu et al. 2003). However, BSEP downregulation may also indicate a direct effect of the tested cholestatic drugs on the regulation of the BSEP transporter, as potent BSEP inhibitors have been shown to downregulate BSEP expression in primary human hepatocytes (Garzel et al. 2014). In addition Kaimal et al. (2009) showed that the potent BSEP inhibitor troglitazone, which acts as FXR modulator, antagonized the bile acid/FXR signaling pathway and significantly repressed bile acid-induced BSEP and SHP expression (Kaimal et al. 2009). Moreover, the decreased expression of FXR (Table 4) can at least partly explain this reduced FXR signaling. This is in line with the finding that both FXR and SHP expressions were reduced by 90 % or more in cholestatic patients (Demeilliers et al. 2006). Thus, based on our findings, it can be postulated that exposure to cholestatic compounds could lead to compromised FXR-mediated adaptive responses, causing hepatic cholestasis. The liver X receptor (LXR) is involved in the regulation of metabolism of lipids and cholesterol to bile acid catabolism. Activation of the LXR receptor is shown to prevent toxicity from bile acid accumulation in female mice (Uppal et al. 2007). We found in human PCLS that the LXR pathway including the genes involved in cholesterol transport such as ABCG5 and ABCG8 was downregulated by the cholestatic drugs, which may indicate a loss of this protective action of LXR. In addition, the PXR and VDR pathways are downregulated due to the exposure of PCLS to the cholestatic drugs. The pregnane X receptor (PXR) is activated by drugs and endogenous molecules and plays a central role in their metabolism by induction of cytochrome P450 enzymes, conjugation enzymes and efflux transporters. PXR activation also regulates bile acid synthesis, their metabolism and transport, cholesterol homeostasis and lipid metabolism. The exposure of the human PCLS to the cholestatic compounds resulted in upregulation of some of the phase I metabolic enzymes such as CYP1A2, CYP2C8 and CYP2C9. However, again in contrast to the results obtained after incubation with 100 μM CDCA, there was no change in the expression of CYP7A1 involved in bile acid synthesis in human PCLS. The vitamin D receptor (VDR) expression is restricted to non-parenchymal cells, such as biliary epithelial cells in the liver (Gascon-Barré et al. 2003). VDR downregulation was not observed in our data, but RXR expression is down regulated. Since VDR acts as a heterodimer with RXR, it can be assumed that VDR function is compromised by this lack of RXR expression. Together, the reduced activation of FXR, LXR, PXR and VDR could be responsible for reduced adaptive responses to the effects of the cholestatic drugs and lead to development of cholestasis.

Oxidative stress is implicated to play a role in the pathogenesis of drug-induced cholestasis as a result of bile acid accumulation (Tanaka et al. 2009; Copple et al. 2010; Anthérieu et al. 2013). Oxidative stress was also shown to play a major role as a primary causal event in the early CPZ-induced cholestasis in human HepaRG cells (Anthérieu et al. 2013). In addition, the protective role of the NRF2-mediated oxidative stress response was reported in mice in response to ANIT-induced cholestasis (Tanaka et al. 2009). This NRF2-mediated oxidative stress response counteracts the cellular oxidative stress by induction of detoxifying and antioxidant enzymes, which also occurred ex vivo as ATF4 (activating transcription factor 4) was upregulated in the human PCLS treated with cholestatic drugs. ATF4 is known to increase the activation of phosphorylated NRF2 protein, key regulator of the oxidative stress response. This indicates that detoxifying mechanisms are activated in the PCLS to alleviate the oxidative stress due to accumulating bile acids. Hepatic fibrosis and hepatic stellate cell activation were also observed in human PCLS due to exposure to the cholestatic drugs. Accumulation of bile acids by obstructive cholestasis (Tag et al. 2015) was shown to lead to an inflammatory response in vivo which in turn leads to activation of hepatic stellate cells and liver fibrosis. We showed recently that rat and human PCLS can be a suitable model to identify the early fibrotic changes and fibrosis-inducing potential of a compound by transcriptomics (Vatakuti et al. 2015) or RT-PCR (van de Bovenkamp et al. 2005; Westra et al. 2014) Early fibrotic response genes identified in rat PCLS such as KLF6 and SERPINE1 were upregulated in human PCLS indicating their prominent role in the early onset of fibrosis. A recent study revealed that endoplasmic reticulum stress is involved in the bile acid-induced hepatocellular injury (Adachi et al. 2014). In PCLS, ER stress, UPR and protein ubiquitination pathways were also among the most affected pathways. The UPR signaling pathway is activated in response to ER stress and promotes cell survival and adaptation. Our results suggest that ER stress, protein ubiquitination and unfolded protein response UPR may be early cellular effects in drug-induced cholestasis.

Cholesterol is the starting material for the synthesis of bile acids in liver. Bile acid biosynthesis is the major catabolic pathway for cholestasis. The genes involved in cholesterol biosynthesis were downregulated in human PCLS indicating the adaptive response of hepatocytes to decrease cholesterol synthesis as a response to cholestatic drugs (supplementary table 2) as was also observed in mouse PCLS (Szalowska et al. 2013). Also, genes in the complement system such as C3 and C5 were downregulated in human PCLS indicating possibly the adaptive response to the inflammation leading to fibrosis. The complement system is known to play a critical role in the pathogenesis of chronic liver disease (Qin and Gao 2006) and is regulated by FXR in both human and rodents (Li et al. 2005). The coagulation system is one of the signaling pathways involved in cellular stress and injury. The coagulation system is known to contribute to bile duct ligation-induced and ANIT-induced liver injury (Abdel-Salam et al. 2005; Luyendyk et al. 2009) in the rat. Downregulation of genes involved in the coagulation system was observed in human PCLS.

### Comparison with human in vivo cholestasis

The results obtained in PCLS should preferably be validated by comparing with human liver tissue of patients suffering from drug-induced cholestasis. However, to the best of our knowledge, this data is not available. Therefore, we compared our findings with gene expression data obtained from liver samples of patients with cholestasis due to biliary atresia and intrahepatic non-drug-induced cholestasis (Bessho et al. 2014). Despite the fact that these samples represent an end-stage disease situation and were mainly obtained from infants, comparison of the activated pathways between human PCLS and the patient samples revealed that there was good overlap with respect to the processes involved in cholestasis, although more pathways were affected in vivo than in the cholestatic PCLS (supplementary Figure 3). The FXR pathway was affected both in vivo and ex vivo; however, in human PCLS, the genes involved in the FXR pathway were downregulated in contrast to the patient samples, where they were upregulated. The genes involved in the LXR pathway and cholesterol biosynthesis were mostly downregulated in both the human PCLS and the patient livers. Remarkably, among the PXR target genes, some of the cytochrome P450 isoforms were strongly downregulated in the human cholestatic livers in vivo but were not regulated or somewhat upregulated in human PCLS. Several genes involved in UPR response were upregulated after exposure to cholestatic drugs in human PCLS but were downregulated in in vivo cholestasis (supplementary Figure 4). Genes involved in the complement system were downregulated in both human PCLS and in patient samples. Liver fibrosis or hepatic stellate cell activation is observed both in human PCLS exposed to cholestatic drugs and in in vivo cholestasis. Structural alteration of tight junction proteins is observed in the bile duct-ligated cholestasis (Fallon et al. 1993). The tight junction signaling pathway is not activated in slices, but significant activation is seen in patient samples (supplementary Figure 3). An explanation for the observed differences between in vivo data and the ex vivo data could be due to the different causes of cholestasis, the age of the patients or the large difference in time frame. In addition, we have performed an analysis of the data of Schröder et al. (2013) and focused on some important genes known to play a role in cholestasis. ABCB11 (BSEP), ABCB4 (MDR3), ABCG5 and NR0B2 (SHP) are downregulated in accordance with the findings in our study. CYP8B1 is downregulated similarly in both studies; CYP7A1 is not regulated in our dataset but surprisingly is upregulated in the dataset of Schroder et al. in contrast to the expected downregulation. In the cholestatic patients, an upregulation of the nuclear receptors NR1H4 (FXR) and NR1H3 (LXR) was observed in contrast to the observed downregulation in human PCLS, which could be due to the different causes of cholestasis or the large difference in time frame. Another reason could be the upregulation of RXRA in cholestatic patients in contrast to downregulation in human PCLS. Nuclear receptors such as FXR and LXR act in combination with RXRA as heterodimers. We observed the downregulation of RXRA in human PCLS across the tested cholestatic drugs and hence possibly explaining the downregulation of nuclear signaling pathways such as FXR and LXR in slices.

## Conclusion

In conclusion, the transcriptomic analysis of human PCLS exposed to cholestatic drugs in the presence of bile acids revealed that this model reflects the primary toxicity processes associated with hepatic cholestasis, and the related processes such as oxidative stress, ER stress and UPR response, and therefore seems to be promising for the future application in drug screening for cholestasis. The results suggest that decreased adaptive responses mediated via nuclear receptors are associated with these cholestatic effects. Our study demonstrates that human PCLS are a suitable model to identify biomarkers and possible mechanisms of toxicity of cholestatic compounds, when incubated in the presence of a physiological concentration of bile acids. However, at this moment, this model cannot be used for screening of large amounts of drug candidates due to the limitation of the availability of fresh human tissue. As soon as successful cryopreservation methods will be established, the availability of the tissue will become much better, and will become similar to that for human hepatocytes. Moreover, for full mechanistic inside on drug-induced cholestasis, additional time points of exposure of the PCLS, more human liver samples and more cholestatic drugs should be tested to better understand the transcriptomic responses with respect to accompanying cholestatic events.

Mechanistic insights such as downregulation of cholesterol biosynthesis, ER stress response and NRF2-mediated oxidative stress response could be included in the adverse outcome pathway of cholestasis.

## Electronic supplementary material

Below is the link to the electronic supplementary material.

Supplementary material 1 (DOCX 1364 kb)

Supplementary material 2 (DOCX 70 kb)
