# Supplementary material for: Validation of precision-cut liver slices to study drug-induced cholestasis: a transcriptomics approach
Source: Arch Toxicol. 2016 Jun 25;91(3):1401–12. doi: 10.1007/s00204-016-1778-8 (PMC5316400; doi:10.1007/s00204-016-1778-8)
Supplement: Supplementary file 1 — Supplementary material 1 (DOCX 1364 kb) [file 204_2016_1778_MOESM1_ESM.docx]

Figure 1: Concentrations selected for gene expression studies and their effect on decrease in cell viability (represented as % of ATP remaining in the slices). Mean values (pmol/µg) and the error bars were derived from 3-5 independent experiments.

Supplementary figure 2


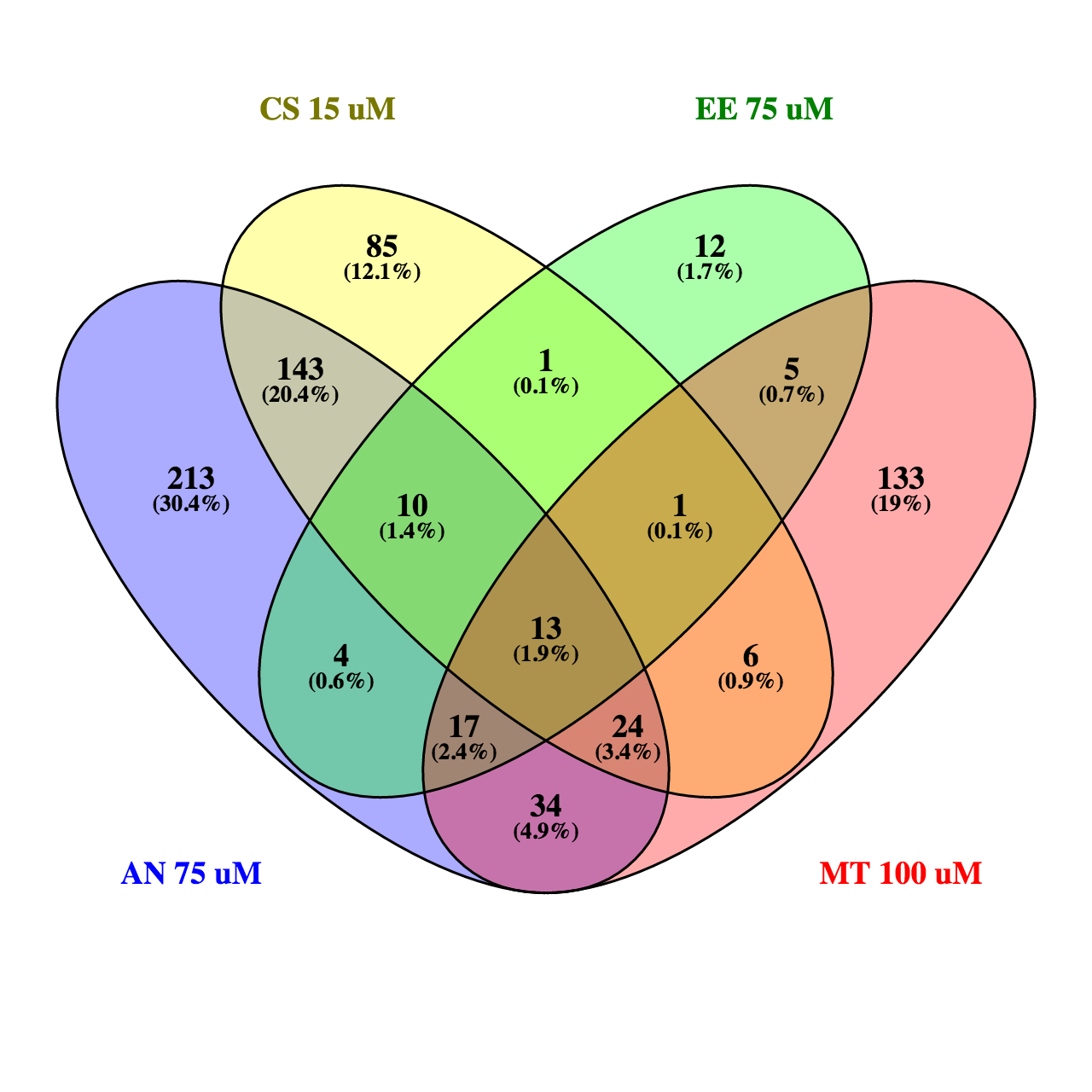


Figure 2: Venn diagram comparison of the number of regulated genes due to cholestatic drugs (The concentration of each compound that induced the higher number of regulated genes was considered for the comparison. (Chlorpromazine (CP 30 μM) was not included in the comparison due to the relatively low number of regulated genes.)


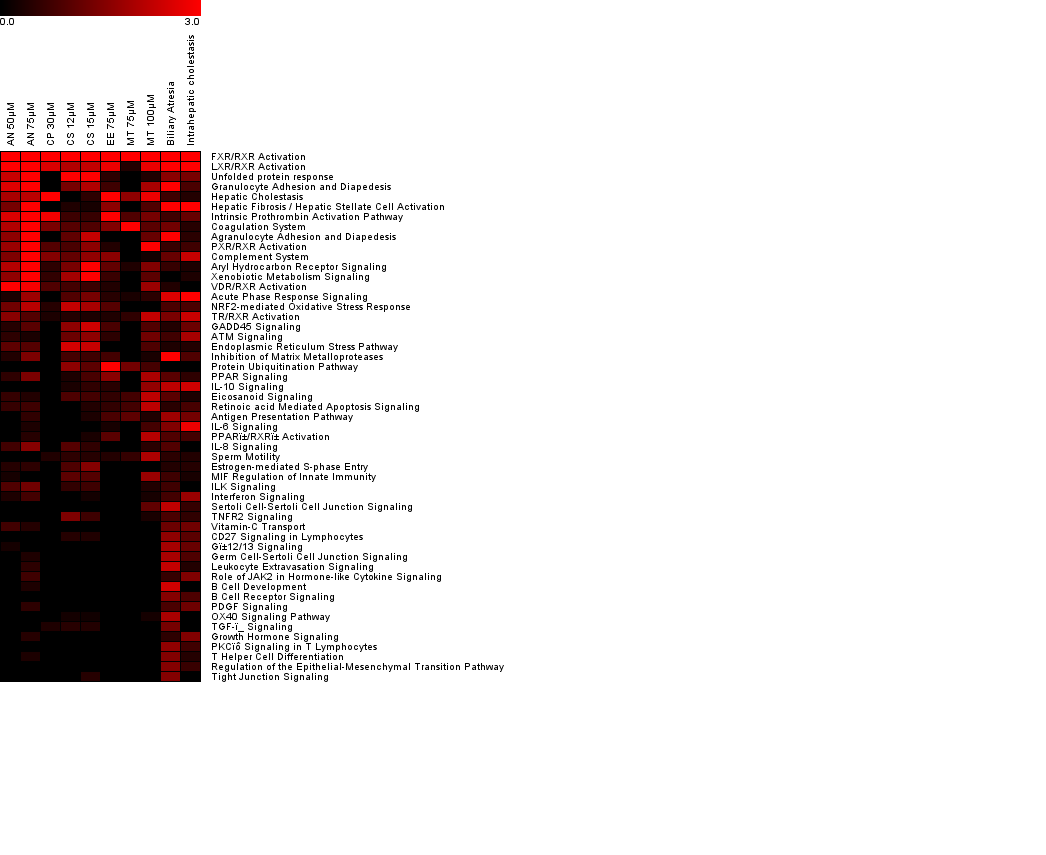


Figure 3: Heatmap of canonical pathway enrichment analysis results. Results from hPCLS were compared with in vivo cholestasis represented by biliary atresia and intrahepatic cholestasis. Enrichment values (-log (p-value)) are scaled from 0 to 3 (black to red).


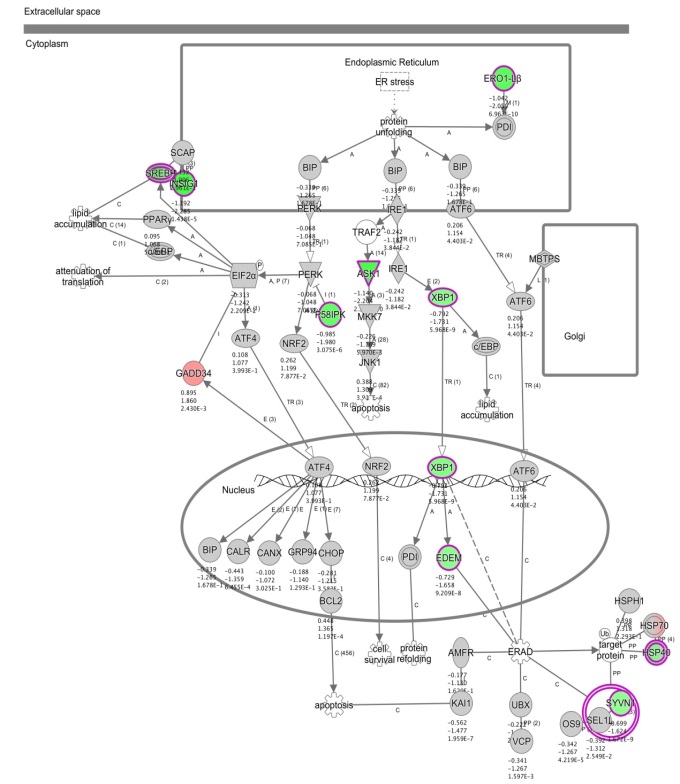

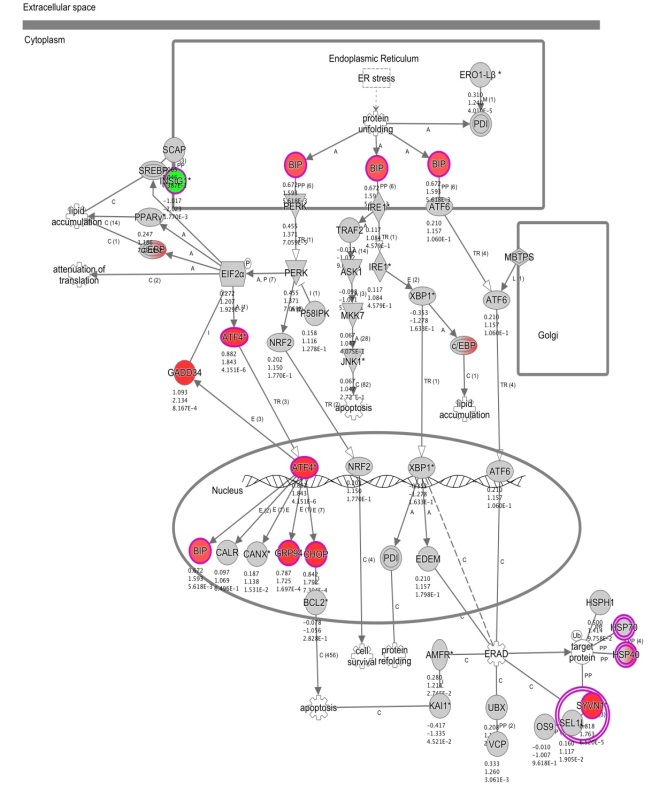


A

B

Figure 4: Unfolded protein response genes regulated due to biliary atresia (A) and cyclosporine 15 µM in human PCLS (B).
