# Supplementary material for: Validation of precision-cut liver slices to study drug-induced cholestasis: a transcriptomics approach
Source: Arch Toxicol. 2016 Jun 25;91(3):1401–12. doi: 10.1007/s00204-016-1778-8 (PMC5316400; doi:10.1007/s00204-016-1778-8)
Supplement: Supplementary file 2 — Supplementary material 2 (DOCX 70 kb) [file 204_2016_1778_MOESM2_ESM.docx]

Table 1: Genes involved in the LXR pathway and their regulation after exposure to cholestatic drugs in human PCLS. Significantly regulated genes with fold change ≤-1.5 or ≥ 1.5 are highlighted in orange and blue respectively.

Table 2: Genes involved in cholesterol biosynthesis and their regulation after exposure to cholestatic drugs in human PCLS. Significantly regulated genes with fold change ≤-1.5 or ≥ 1.5 are highlighted in orange and blue respectively.

Table 3: Genes involved in the PXR pathway and their regulation after exposure to cholestatic drugs in human PCLS. Significantly regulated genes with fold change ≤-1.5 or ≥ 1.5 are highlighted in orange and blue respectively.

Table 4: Genes involved in the VDR pathway and their regulation after exposure to cholestatic drugs in human PCLS. Significantly regulated genes with fold change ≤-1.5 or ≥ 1.5 are highlighted orange and blue respectively.

Table 5: Genes involved in the complement system and their regulation after exposure to cholestatic drugs in human PCLS. Significantly regulated genes with fold change ≤-1.5 or ≥ 1.5 are highlighted in orange and blue respectively.

Table 6: Genes involved in the coagulation system and their regulation after exposure to cholestatic drugs in human PCLS. Significantly regulated genes with fold change ≤-1.5 or ≥ 1.5 are highlighted in yellow and blue color respectively.

Table 7: NRF2 mediated oxidative stress response genes and their regulation after exposure to cholestatic drugs in human PCLS. Significantly regulated genes with fold change ≤-1.5 or ≥ 1.5 are highlighted in orange and blue color respectively.

Table 8: Genes involved in hepatic fibrosis/ hepatic stellate activation and their regulation after exposure to cholestatic drugs in human PCLS. Significantly regulated genes with fold change ≤-1.5 or ≥ 1.5 are highlighted in orange and blue respectively.
